# Unraveling the link between family of origin and parental responsiveness toward own child

**DOI:** 10.3389/fpsyt.2023.1255490

**Published:** 2023-10-09

**Authors:** Paulina Anikiej-Wiczenbach, Maria Kaźmierczak

**Affiliations:** Institute of Psychology, Faculty of Social Sciences of the University of Gdansk, Gdańsk, Poland

**Keywords:** family of origin, parental responsiveness, Ainsworth Scale, observational measures, family dynamics

## Abstract

This study investigates the influence of family of origin on parental responsiveness toward own child, taking into account gender differences. A total of 110 triads of mothers, fathers, and their first child aged 6–10 months participated in the standardized Free Play procedure. Parental responsiveness was assessed through observational measures (using Ainsworth procedure) and self-reported scales (Parental Responsiveness Scale). Results revealed correlations between objectively assessed responsiveness and self-reported parental styles in the family of origin, separately for mothers and fathers. Among mothers having daughters, parental sensitivity (an important aspect of observationally measured responsiveness) was positively correlated with having had a liberal loving mother and a negative correlation with an autocratic mother. Cooperation (another aspect of observationally measured responsiveness) was correlated positively with having had a liberal loving mother. Meanwhile, having a liberal unloving mother predicted lover sensitivity and cooperation. Similar correlations were not observed for mothers having sons. Among fathers having daughters, both aspects of observed responsiveness were positively correlated with having had a democratic father and negatively with autocratic or liberal unloving parents. Moreover, having a liberal unloving father and autocratic mother predicted their lower responsiveness toward daughters. These findings highlight the role of family dynamics in shaping parental responsiveness and emphasize the importance of understanding these dynamics in promoting responsive parenting.

## 1. Introduction

Parental responsiveness can be defined as the extent to which parents appropriately and promptly respond to their child's needs and signals. Parents who are responsive can create a positive emotional climate in the home, which can help children feel loved, valued, and supported (1). This can lead to better mental health outcomes for children (2), including lower levels of anxiety and depression, and can model positive social and emotional behaviors. This can help children develop important skills such as empathy, self-regulation, and social problem-solving (3–5). There is a dearth of adequate methods for assessing parental responsiveness, as well as a scarcity of studies establishing comprehensive connections between self-reported measures and observational measures of parental reactions.

Research suggests that experiences from one's family of origin shape the way one builds a partnership in one's family of procreation (6) and can influence the way parents interact with their children (7). Specifically, individuals who were raised in households with warm, supportive, and responsive parents may be more likely to exhibit similar parenting behaviors with their own children (3). On the other hand, individuals may deliberately choose to adopt a different parenting style than the one they experienced in their own childhood, based on their own beliefs, values, and experiences. For example, someone who was raised in an authoritarian household may choose to adopt a more democratic parenting style because they believe it will be more effective in promoting their child's development and wellbeing. Autocratic and permissive styles turn out to be connected to higher levels of externalizing difficulties, while the authoritative style has been linked to lower levels of these problems in children (8).

According to Ainsworth et al. (1), two aspects of responsiveness can be analyzed: (1) sensitivity toward the child's behaviors (being aware of what the child needs and reacting to these needs with tenderness) and (2) cooperation with the child's behaviors (giving the child space for exploration and paying attention to the child's interests, not interfering, and not introducing one's own programs). Moreover, one of the key responsive behaviors is emotional availability, which refers to the ability of parents to be attuned to their children's emotional needs and to respond in a supportive and sensitive way (9). Parents who are emotionally available are able to validate their children's feelings, offer comfort and support, and help their children regulate their emotions (10). Providing prompt, adequate, and loving responses to a child's cues can help children develop greater empathy, self-regulation, social problem-solving, and self-esteem, and helps them learn how to engage in healthy relationships in the future (11).

Parents can draw their styles of parenting from their experiences in their family of origin. Most current parents in Poland grew up in post-communist homes, where the understanding of parenthood was significantly different from today's approach (12). Recent research suggests that Polish couples face a dilemma between the traditional ideal of heavily engaging in childcare, consistent with the image of the “Polish mother,” and the egalitarian image of marriage. According to the traditional model, a strong woman balances family and work obligations (an ideal deemed crucial to family functioning and natural for a woman in the communist era) and she must make many difficult sacrifices; meanwhile, the father is the breadwinner, always absent (13). However, nowadays the egalitarian image of marriage (with both partners engaged in family and work roles) has become more and more popular, especially among well-educated couples with stable financial situations (14).

Referring to the abovementioned sociocultural influences differences in relationships of both parents parental styles with their daughters and sons should be noted. A study found that sons are raised with more permissive parenting attitudes than are daughters (15). The mother's parenting style may have a greater impact on the mental health of emerging adult daughters than sons (7). Furthermore, studies suggest that sons have increased externalizing problems linked to decreased paternal control, negative perceptions of their mothers, and lower emotional availability of both parents, whereas daughters have increased externalizing problems linked to increased maternal control and paternal psychopathology (16). One study found that sons are raised with more permissive parenting attitudes than are daughters (17). Furthermore, the mother's parenting style may have a greater impact on the mental health of emerging adult daughters than sons (7).

Based on theory of family systems, there are four parenting styles: (1) democratic, (2) autocratic, (3) liberal loving, and (4) liberal unloving (18). In the democratic style, parents respect the child's rights, trust them, and provide friendship and kindness. Parents are responsive to their children's needs, respect them, and react appropriately, giving to the child what the child desires, or providing an explanation as to why they cannot get what they want. In autocratic families, parents are the most powerful family members and they establish the duties and rights of children. Such parents are not responsive to the child's needs. Instead of warmth and empathy they are inclined to detect and criticize the child's errors and control the way the child learns. In families displaying the liberal style, parents do not interfere with the child's behaviors and leave the child completely free. They show interest in the child only when the child expects and demands it. In the loving form, parents provide the child with tenderness and love and they believe that leaving the child alone to explore the world is the best option. In the unloving form, they are indifferent to the child, display emotional coldness, and lack interest in the child's life.

The aim of this study was to explore the links between these parental styles and observed and self-reported responsiveness toward one's own child. The question arises whether parental styles in family of origin can affect parental responsiveness to one's own child. We hypothesize that both mothers and fathers raised by more democratic parents will present higher levels of responsiveness than mothers and fathers raised by autocratic or liberal parents. We expect also that having experienced liberal loving parenting in one's family of origin will be connected with higher levels of cooperation with child's behaviors. Regarding the cooperation, we expect that it will be higher in parents, who were raised by liberal loving caregivers. This because they raised in the environment where their interest to different activities was reinforced and their autonomy was respected. Additionally, we will explore gender differences in parental responsiveness.

To answer these questions, mothers and fathers of young children (aged 6–9 months) rated their own parents (mothers and fathers separately) according to their past experiences in their family of origin.

Then they participated in a Free Play procedure with their child and filled out the Parental Responsiveness Scale (self-reported responsiveness). Their interactions with the child during the Free Play were recorded and then judged by trained raters using the Ainsworth Sensitivity and Cooperation Scale (objective responsiveness). This approach allowed us to explore the construct of parental responsiveness over a long period of time, taking into account socio-cultural and gender perspectives.

## 2. Methods

A standardized procedure was designed to mitigate the influence of external factors in the study. The research took place in a laboratory resembling a child's room, equipped with a one-way mirror. Within the room, two cameras were positioned to capture a comprehensive view of the interactions. This setup enabled real-time observation of the parent–child interaction. Additionally, the cameras recorded the session, facilitating a thorough assessment of every aspect of the parent–child interaction.

The free play session lasted for 10 min, during which parents were instructed to engage with their children as they typically would. The laboratory was arranged identically for each session, with every parent receiving an identical set of toys such as rattles, books, and animal figurines. While one parent interacted with the child, the other parent completed a series of questionnaires in a separate room adjacent to the examination room. Then they switched roles.

### 2.1. Measures

#### 2.1.1. Parental sensitive responsiveness

Parental Responsiveness was evaluated using both observational and self-reported measures. For the observational assessment, the researcher analyzed recorded footage from the free-play procedure to assess parental sensitivity toward their own child. The Ainsworth Scale for Sensitivity and Cooperation (1) was used to assign scores to parental behaviors. Scores on this scale range from one to nine points, with higher scores indicating higher levels of Sensitivity and Cooperation. The Sensitivity vs. Insensitivity Subscale, part of the Ainsworth Scale, measures a parent's ability to accurately interpret signals from the child, identify the infant's implicit attitude, and respond appropriately and promptly. Similarly, the Cooperation vs. Interference With Baby's Ongoing Behavior Subscale, also on a nine-point scale, assesses the extent to which parental interventions interrupt or curtail the baby's activities vs. responding adequately and in a timely manner to the baby's state, mood, and interests. This subscale evaluates physical interference with the baby's current activity and the frequency of interruptions. To ensure consistency and avoid bias, individuals from the same family were coded separately. Furthermore, a 30% overlap was utilized to assess the reliability of the coders' evaluations.

In addition to the observational measures, parents themselves assessed their own responsiveness to their infant's cues using the seven-point Parental Responsiveness Scale (PRS) (19), which consists of 13 statements. In this study, the PRS demonstrated good reliability and internal and external accuracy, with a Cronbach's alpha coefficient of 0.81 for women and 0.82 for men.

#### 2.1.2. Experiences in a family of origin

The Family of Origin scale (18) was used. Participants asses retrospectively the behaviors of their mother and father on a five-point scale. The scale contains 34 statements, that pertain to various aspects, including emotional support, the system of rewards and penalties, display of affection, communication between child and parent, and control of the child's behavior. The tool is composed of four subscales indicating parental styles (judged retrospectively) democratic, autocratic, liberal loving and liberal unloving, each of them composed by 10 items (some of items are used for more than one subscale ex. “In my family, my mother tried—to the best of her ability—to meet the needs of all family members.”—is included in subscales for democratic and liberal loving mother). The Cronbach's alpha coefficient in this study was presents satisfactory reliability for all subscales (1) democratic mother 0.90 for women and 0.80 for men; (2) autocratic mother 0.88 for women and 0.76 for men; (3) liberal loving mother 0.67 for women and 0.68 for men; (4) liberal unloving mother 0.84 for women and 0.90 for men; (5) democratic father 0.92 for women and 0.90 for men; (2) autocratic father 0.89 for women and 0.86 for men; (3) liberal loving father 0.63 for women and 0.68 for men; (4) liberal unloving father 0.88 for women and 0.92 for men.

### 2.2. Participants

A total of 110 triads (mother, father, and their first and only child; *N* = 330) participated; the children were aged from 6 to 10 months (*M* = 7.49; *SD* = 1.15), including 49 (44.55%) parents of boys. The children had been born between 37 and 42 weeks (*M* = 39.95; *SD* = 1.30). Most of them were born through vaginal delivery (78; 70.3%) and the rest of them were born by cesarean section. The exclusion criteria were diseases and developmental abnormalities. The mothers were aged 20–41 years (*M* = 29.91; *SD* = 3.62) and the fathers were aged 25–50 years (*M* = 31.2; *SD* = 3.77). They had been in close relationships for from 1 to 23 years (*M* = 7.47; *SD* = 3.99). The majority of mothers had higher education (*n* = 91), some of them had secondary education (*n* = 11) and vocational education (*n* = 7), and one of them had primary education (*n* = 1). The majority of women were working (*n* = 95; 85.6%). A total of 91 parents were married (82%) and the rest of them were cohabiting; 81% of families were living in a city and the rest of them were living in the countryside. Parents were recruited in antenatal schools in the Pomeranian area and through advertisements on social media.

SPSS 27.0 was used to calculate the means, standard deviations, and Pearson correlation coefficients.

## 3. Results

The correlations of self-reported and objectively judged parental responsiveness (sensitivity and cooperation) and parental styles in the family of origin are presented in Tables 1, 2 for women, and Tables 3, 4 for men.

**Table 1:** Correlation coefficients between variables for mothers of girls.

**Variable**	**1**.	**2**.	**3**.	**4**.	**5**.	**6**.	**7**.	**8**.	**9**.	**10**.	**11**.
1. Parental responsiveness—sensitivity	—										
2. Parental responsiveness—cooperation	0.92^**^	—									
3. Parental responsiveness—PRS	0.33^*^	0.23^†^	—								
4. Democratic mother	0.20	0.13	0.28^*^	—							
5. Autocratic mother	−0.27^*^	−0.22^†^	−0.21	−0.86^**^	—						
6. Liberal loving mother	0.31^*^	0.27^*^	0.23^†^	0.83^**^	−0.83^**^	—					
7. Liberal unloving mother	−0.23^†^	−0.23^†^	−0.14	−0.82^**^	0.81^**^	−0.76^**^	—				
8. Democratic father	0.13	0.03	0.26	0.49^**^	−0.43^**^	0.52^**^	−0.31^*^	—			
9. Autocratic father	−0.10	0.01	−0.32^*^	−0.45^**^	0.48^**^	−0.42^**^	0.37^**^	−0.77^**^	—		
10. Liberal loving father	0.21	0.10	0.29^*^	0.43^**^	−0.45^**^	0.58^**^	−0.33^*^	0.88^**^	−0.75^**^	—	
11. Liberal unloving father	−0.07	0.05	−0.26^†^	−0.40^**^	0.39^**^	−0.43^**^	0.21	−0.90^**^	0.79^**^	−0.79^**^	—

^†^*p* < 0.10; ^*^p < 0.05; ^**^p < 0.01.

**Table 2:** Correlation coefficients between variables for mothers of boys.

**Variable**	**1**.	**2**.	**3**.	**4**.	**5**.	**6**.	**7**.	**8**.	**9**.	**10**.	**11**.
1. Parental responsiveness—sensitivity	—										
2. Parental responsiveness—cooperation	0.92^**^	—									
3. Parental responsiveness—PRS	0.05	−0.02	—								
4. Democratic mother	−0.11	−0.16	0.12	—							
5. Autocratic mother	−0.05	0.07	−0.11	−0.77^**^	—						
6. Liberal loving mother	−0.11	−0.21	0.10	0.83^**^	−0.64^**^	—					
7. Liberal unloving mother	0.07	0.15	−0.18	−0.84^**^	0.76^**^	−0.61^**^	—				
8. Democratic father	−0.24	−0.13	0.26^†^	0.22	−0.11	0.10	−0.08	—			
9. Autocratic father	0.09	0.02	−0.18	−0.24	0.23	−0.19	0.15	−0.53^**^	—		
10. Liberal loving father	0.18	−0.10	0.30^*^	0.26^†^	−0.08	0.18	−0.06	0.89^**^	−0.46^**^	—	
11. Liberal unloving father	0.16	0.06	−0.19	−0.22	0.19	−0.03	0.21	−0.65^**^	0.82^**^	−0.45^**^	—

^†^*p* < 0.10; ^*^p < 0.05; ^**^p < 0.01.

**Table 3:** Correlation coefficients between variables for fathers of girls.

**Variable**	**1**.	**2**.	**3**.	**4**.	**5**.	**6**.	**7**.	**8**.	**9**.	**10**.	**11**.
1. Parental responsiveness—sensitivity	—										
2. Parental responsiveness—cooperation	0.93^**^	—									
3. Parental responsiveness—PRS	0.18	0.12	—								
4. Democratic mother	0.21	0.19	0.28^*^	—							
5. Autocratic mother	−0.36^**^	−0.32^*^	−0.21	−0.54^**^	—						
6. Liberal loving mother	0.16	0.15	0.22	0.80^**^	−0.39^**^	—					
7. Liberal unloving mother	−0.36^*^	−0.30^*^	−0.16	−0.65^**^	0.79^**^	−0.40^**^	—				
8. Democratic father	0.30^*^	0.31^*^	−0.02	0.09	−0.14	0.05	−0.11	—			
9. Autocratic father	−0.29^*^	−0.27^*^	−0.18	−0.02	0.11	0.01	0.14	−0.53^**^	—		
10. Liberal loving father	0.20	0.25^†^	0.02	0.05	−0.13	0.17	0.02	0.85^**^	−0.38^**^	—	
11. Liberal unloving father	−0.31^*^	−0.33^*^	−0.02	−0.06	0.11	0.01	0.12	−0.68^**^	0.69^**^	−0.40^**^	—

^†^*p* < 0.10; ^*^p < 0.05; ^**^p < 0.01.

**Table 4:** Correlation coefficients between variables for fathers of boys.

**Variable**	**1**.	**2**.	**3**.	**4**.	**5**.	**6**.	**7**.	**8**.	**9**.	**10**.	**11**.
1. Parental responsiveness—sensitivity	—										
2. Parental responsiveness—cooperation	0.92^**^	—									
3. Parental responsiveness—PRS	0.17	0.27^†^	—								
4. Democratic mother	0.07	0.09	0.31^*^	—							
5. Autocratic mother	−0.03	−0.14	−0.36^*^	-0.65^**^	—						
6. Liberal loving mother	0.17	0.13	0.29^†^	0.68^**^	−0.41^**^	—					
7. Liberal unloving mother	0.02	0.02	−0.29^†^	−0.66^**^	0.64^**^	−0.08	—				
8. Democratic father	0.13	0.15	0.06	0.30^*^	−0.27^†^	0.01	−0.31^*^	—			
9. Autocratic father	−0.01	−0.04	−0.05	−0.24	0.22	0.01	0.31*	−0.69^**^	—		
10. Liberal loving father	0.13	0.14	−0.02	0.31^*^	−0.36^*^	0.17	0.23	0.81^**^	−0.69^**^	—	
11. Liberal unloving father	−0.14	−0.13	−0.19	−0.18	0.10	0.06	0.30^*^	−0.80^**^	0.74^**^	−0.58^**^	—

^†^*p* < 0.10; ^*^p < 0.05; ^**^p < 0.01.

We found that, among mothers who have daughters, there was a positive correlation between sensitivity (an objectively measured aspect of responsiveness) and having had a liberal loving mother in their own family of origin (see Table 1). Conversely, there was a negative correlation between sensitivity and having had an autocratic mother in their family of origin. Furthermore, among mothers with daughters, there was also a positive correlation between cooperation (another objectively assessed aspect of parental responsiveness) and having had a liberal and loving mother in their own family of origin.

Mothers who had daughters had a positive correlation of self-reported responsiveness with having had a democratic mother, a liberal and loving father, and a negative correlation of self-reported responsiveness with having had an autocratic father. Surprisingly, aside from the positive correlation with having had a liberal and loving father, these correlations were not observed in mothers who had sons (see Table 2).

We also found that, among fathers who have daughters, there was a positive correlation between both aspects of observational responsiveness (sensitivity and cooperation) and having had a democratic father in their own family of origin (see Table 3). Conversely, there was a negative correlation between both aspects of responsiveness (sensitivity and cooperation) and having had an autocratic or liberal unloving mother or father in the family of origin. There were no correlations between observational responsiveness and any aspects of parental styles in family of origin among fathers who had sons (see Table 4).

Fathers who had daughters showed a positive correlation of self-reported responsiveness with having a democratic mother and fathers who have sons also showed a positive correlation of self-reported responsiveness with having had a democratic mother and a negative correlation with having had an autocratic mother.

To respond to such a question whether the parental styles in family of origin are predictors of parental responsiveness a linear regression analysis was performed. As predictors for women's sensitivity toward own daughters, the autocratic and liberal loving maternal styles judged retrospectively were used. The model proved to fit the data and explained 10% of the variance of women's sensitivity: *R*^2^ = 0.10; *F*_(1, 56)_ = 6.01; *p* = 0.017. Only liberal loving maternal style judged retrospectively (β = 0.31, *p* = 0.001) predicted higher women's sensitivity. Similar results were obtained for the women's cooperation with own daughters. The model explained 7% of the variance [*R*^2^ = 0.07; *F*_(1, 57)_ = 4.46; *p* = 0.003] and having liberal loving mother predicted higher cooperation to own daughter (β = 0.27, *p* = 0.039). Meanwhile, as predictors for men's sensitivity toward own daughters the autocratic maternal and democratic, autocratic, liberal unloving paternal styles judged retrospectively were tested. The results showed that the model fit to data and explain 20% of variance of men's sensitivity [*R*^2^ = 0.20; *F*_(2, 55)_ = 6.73; *p* = 0.002]. An autocratic maternal style (β = −0.32, *p* = 0.013) and liberal unloving paternal style (β = −0.27, *p* = 0.031) judged retrospectively predicted lower men's sensitivity. For the men's cooperation with own daughters, the model explained 18% of the variance [*R*^2^ = 0.18; *F*_(2, 56)_ = 6.30; *p* = 0.003] and the autocratic mother (β = −0.29, *p* = 0.020) and liberal unloving father (β = −0.29, *p* = 0.021) in family of origin predicted lower men's cooperation toward own daughters.

## 4. Discussion

The present study investigated the correlations between objectively judged parental responsiveness (measured with an observational scale), self-reported responsiveness, and parental styles in the family of origin among mothers and fathers of young children (6–9 months). The findings shed light on the complex interplay between family dynamics and parental behaviors. The results confirmed that patterns from the family of origin are important for parent–child relationships.

The study revealed that there are different associations between observational and self-reported responsiveness, as well as measures of parental attitudes in the family of origin. This suggests that some parental behaviors from the family of origin are more likely to be repeated in one's own family, even at the very early stages of parenthood. Conversely, certain parental behaviors from the family of origin influence one's judgement of one's own behaviors [also in terms of parenting; (8)]. For instance, having a liberal and loving parent in the family of origin may provide individuals with more space to explore and develop their own strategies in stressful situations, such as becoming a first-time parent. The findings suggest that the influence of parental behaviors from the family of origin on one's own parenting practices can be diverse. However, it is important to consider the cultural context, such as parenting in Polish culture and the societal changes that have occurred over the past few decades.

In the context of Polish culture, traditional family values and hierarchical parenting styles have been prevalent in the past. According to the traditional model of parenthood in Poland, mothers should be the primary caregivers, and they are expected to sacrifice themselves to raise a healthy and strong child and to foster a warm family environment (13). This could be a reason why, in the self-reported scale, they judged themselves higher than men, which is not so obvious according to observational measures. However, societal changes have brought about shifts in parenting practices. In recent years, there has been a growing emphasis on democratic and child-centered and responsive parenting approaches (20). Moreover, traditional gender roles, where mothers were primarily responsible for childcare and household duties, are gradually evolving. More fathers are now actively involved in parenting and sharing responsibilities with mothers, contributing to a more balanced and equal division of care giving tasks (20). Moreover, the correlation between mothers' and fathers' self-reported responsiveness suggests that parents are usually congruent with the behaviors they display toward their own child. Women may help men adapt to the role of being a father (21), and indeed, the participation of fathers in the lives of their children has increased in the last two decades (22).

The study's findings suggest that individuals who grew up with liberal and loving parents in the family of origin may have had more opportunities to develop their own unique parenting strategies, drawing from the changing cultural norms and values in Polish society.

Furthermore, women who perceived their mother and father as liberal and loving exhibited more positive behaviors toward their own children of the same gender. Conversely, women who rated their mothers as autocratic presented more negative behaviors toward their own daughters. This suggests also that mothers who have experienced a nurturing and supportive maternal figure in their own upbringing are more likely to exhibit sensitive responses toward their daughters. Mothers who perceive their mother as having been authoritarian may struggle to exhibit sensitive responses toward their daughters. Indeed, much research emphasizes the impact of a mother's relations with her mother on the well-being and mental health of her daughters [e.g., (23)].

Regarding self-reported responsiveness, mothers who had daughters demonstrated positive correlations with having had a democratic mother and a liberal loving father, as well as a negative correlation with having had an autocratic father. There was also a positive correlation between a having father with the liberal loving style and self-reported responsiveness to one's own son. These correlations were based on retrospective judgments, suggesting that mothers' perceptions of their own responsiveness are connected with perceived parenting styles in their family of origin.

Previous research suggests that having had a democratic mother promotes prosocial emotions and better emotional regulation (24). Indeed, emotional regulation is crucial for responsiveness and empathy [i.e., the presence of empathic concern and perspective taking, not focused on personal distress; (10)].

The results show that, among fathers of daughters, both aspects of observational responsiveness (sensitivity and cooperation) were positively correlated with the perception of their fathers in their family of origin as democratic. Conversely, negative correlations were found between both aspects of responsiveness and perceiving their mother as autocratic or liberal unloving. These results suggest that fathers who grew up in less caring or authoritarian households may find it difficult to show their daughters sensitivity and cooperation. Interestingly, no correlations were observed between observational responsiveness and any aspects of parental styles in the family of origin among fathers who have sons.

Similar to mothers, self-reported responsiveness among fathers who have both daughters and sons showed positive correlations with having had a democratic mother. Meanwhile, negative correlations were observed with having had an autocratic mother in fathers of boys. Furthermore, there was a positive correlation among fathers of daughters with having had a democratic father. Indeed, research shows that having warm, supportive parents and low hostility home in one's family of origin is positively linked with how children later behave in intimate relationships (25).

It is worth mentioning that only negative parenting styles within the family of origin predict parental responsiveness. For women, having a liberal, unloving mother predicts lower sensitivity and cooperation with their own daughters, while for men, having a liberal, unloving father and an autocratic mother predicts lower sensitivity and cooperation with their daughters. Indeed, children whose parents limit their autonomy, fail to support their goals, and withhold warmth and acceptance tend to be less psychologically resilient (26). They encounter difficulties in pursuing their own objectives and often resort to avoidance strategies when faced with new situations and challenges in adulthood. Moreover, they also lack adequate role models from whom they can learn sensitive responses to child cues (26). However, in men, the prediction of responsiveness by parental styles in the family of origin is stronger than in women, which suggests that women may face different social expectations or possess different personal predispositions for parenting. Nevertheless, the results indicate that parental responsiveness is a complex construct that should also be considered in the context of other variables, such as empathy or attachment (27).

Overall, the results of this study underscore the importance of considering both objective and subjective measures of parental responsiveness and the impact of family of origin on these behaviors. Some studies suggest that the gender of the child plays a key role in the way in which parents behave toward them. There are differences in reading books with one's own child [parents use higher proportions of science talk with daughters than sons; (28)], on their experience of fear [maternal implicit gender stereotypes are associated with daughters' experiences of fear; e.g., (29)], and so on. Furthermore, studies suggest that these parenting styles in one's family of origin mainly influence daughters [e.g., (7)]. However, some studies do report effects on sons (15).

The findings suggest that there are differences between raising sons and daughters and that these differences can be related to a parent's image of their own parents [e.g., (7)].

The observational dimensions of responsiveness—sensitivity and cooperation—were highly correlated with each other, but the parental responsiveness measured with self-reported tool was not always correlated with these observational dimensions. This suggests that measures of responsiveness (self-report and observational) are not congruent and probably depend on other variables. The subscales indicating the parental styles in the family of origin were highly correlated with each other. There was a positive correlation between democratic and liberal loving styles, and between autocratic and liberal unloving styles. There were also negative correlations between democratic and both liberal unloving and autocratic styles, as well as between liberal loving and both liberal unloving and autocratic styles, in almost every aspect across all variants of research groups (mothers having daughters, mothers having sons, etc.). The differences between these parenting styles stem from the combination of control, love, respect, and autonomy. The thing that mostly distinguishes these styles is the tenderness and love provided to the child. In both democratic and liberal loving parenthood, the most important thing for parents is the child's best interest, even if they demonstrate this in different ways. In the autocratic parenting style, the parent may want to raise their child in the way they believe is good for them, but they use less empathy and warmth in their methods and demonstrates the lack of respect. The same lack of the respect is in liberal-unloving style, but additionally parent is not interested in the caregiving and educating own child. Meanwhile in the democratic styles a respect for the child or his needs and he is interesting in parenting.

This study attempted to comprehensively explain the complex mechanisms that start in a parent's family of origin and present in the interaction with their child. However, it is important to highlight some limitations. The potential bias of the sample toward parents who are interested in parenting is a common difficulty in studies such as this. The results' interpretation is constrained by the fact that all measurements were completed in a single visit. Nevertheless, the study's multimethod approach stands out as a major merit—even though the situational setting was restricted to the laboratory room, we were able to conduct measurements based on actual interactions that appeared during free play between the parent and child. In an unfamiliar scenario, parents could explain how they care for their child. It is worth emphasizing that the current study is one of few that incorporates both observational and self-report measures.

The findings suggest that the quality of relationships with parents in one's family of origin can have lasting effects on parental responsiveness, albeit with differences observed between mothers and fathers and based on the gender of the child. These results contribute to our understanding of the complex dynamics of parenting and emphasize the need for further research to explore additional factors that may mediate or moderate these relationships, as well as their implications for child development and family interventions.

## Data availability statement

The data that support the findings of this study are available from the corresponding author upon reasonable request.

## Ethics statement

The studies involving humans were approved by Ethics Committee at the Institute of Psychology, University of Gdansk, Poland (permission #4/2016 and permission#6/2018). The studies were conducted in accordance with the local legislation and institutional requirements. Written informed consent for participation in this study was provided by the participants' legal guardians/next of kin.

## Author contributions

PA-W: Conceptualization, Data curation, Formal analysis, Funding acquisition, Investigation, Methodology, Project administration, Resources, Software, Validation, Visualization, Writing—original draft, Writing—review and editing. MK: Conceptualization, Data curation, Formal analysis, Funding acquisition, Investigation, Methodology, Supervision, Project administration, Resources, Software, Validation, Visualization, Writing—original draft, Writing—review and editing.
